# Thymic Carcinoma Treated by CyberKnife Stereotactic Body Radiotherapy

**DOI:** 10.7759/cureus.1056

**Published:** 2017-02-26

**Authors:** Yuko Harada, Shinichiro Miyazaki

**Affiliations:** 1 Internal Medicine, Shin-yurigaoka General Hospital; 2 Radiation Oncology, Shin-yurigaoka General Hospital

**Keywords:** cyberknife radiosurgery, thymic carcinoma, sbrt

## Abstract

The standard treatment for advanced thymic carcinoma has not yet been established. Most patients have no symptoms until the advanced stage. Radiation therapy has been used for advanced stage cancer, usually in combination with surgery or chemotherapy; however, the survival rates are 30%-50%. We performed hypofractionated stereotactic radiotherapy with CyberKnife (Accuray, Sunnyvale, CA, USA) for 10 cases of advanced thymic cancer. All cases reached at least partial remission (PR) in two months with progression-free irradiated lesions and minimal radiation-related toxicity. It took only seven to 12 days for each therapy that did not require admission. CyberKnife is beneficial for patients even at the terminal stage.

## Introduction

Thymic carcinoma is a rare mediastinal neoplasm with poor prognosis. Engel and Pfeiffer reported that the overall incidence of malignant thymoma was 0.15 per 100,000 person-years in the United States and that it was highest among Asians/Pacific Islanders (0.49 per 100,000 person-years) [1]. In Europe, the incidence of epithelial tumour of the thymus is 1.7 per million per year, and it is higher in central and southern Europe [2-3]. Five-year survival rates are approximately 30%-50%, and optimal management of thymic carcinoma has yet to be defined [4]. Surgery is the mainstay of treatment, and systemic chemotherapy represents the standard of care for metastatic or inoperable refractory/recurrent disease; however, there remains a lack of standard treatment after first-line failure [5]. Radiation therapy has been used in combination with surgery and chemotherapy, as Ogawa, et al. reported; multimodal treatment of complete resection and postoperative radiotherapy with or without chemotherapy is a curative therapy [6]. Most of the previous reports are those of conventional radiation therapy. In the clinical analysis of 45 patients, the three-year survival rate was 59.4% in patients treated with conventional radiotherapy and 80% in those treated with stereotactic body radiotherapy (SBRT) [7]. In the literature, there is only one case report of SBRT with CyberKnife (Accuray, Sunnyvale, CA, USA) [8]. Here, we report our experience of SBRT with CyberKnife for 10 cases of advanced thymic carcinoma.

Informed consent was obtained from all patients for this study.

## Materials and methods

**Figure 1:**
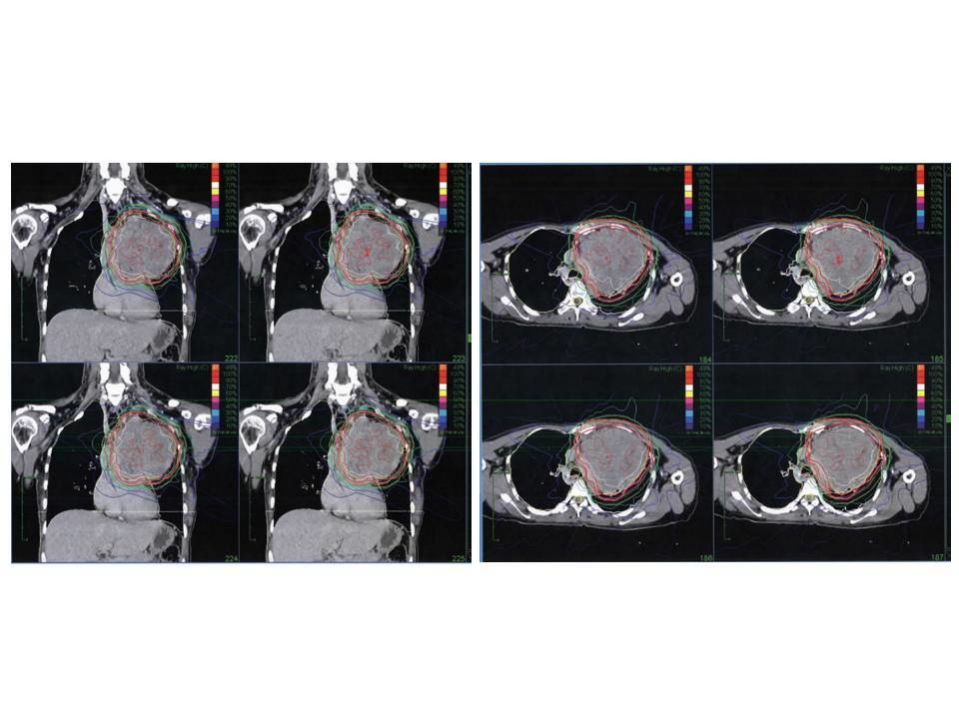
Treatment plan for Case 4

Ten patients were enrolled in the study as can be seen in Table 1. Thymic carcinoma had been pathologically confirmed. All of the patients were treated with CyberKnife. Masaoka stage criteria were used to categorize the patients. Half of the patients (Cases 1 to 5 in Table 1) had undergone radiation therapy for the primary tumor. The other half of the patients (Cases 6 to 10) had had radiation therapy for recurrent or metastatic disease. The prescription dose was calculated according to the size of the tumor with adjustments to minimize the damage to surrounding organs, as seen in Figure 1. For the primary tumor, it ranged from 3100 to 5000 cGy with fractions of 7 to 12. Using α/β ratio in the linear quadratic model of 10 Gy, the doses were calculated to be 35-63 Gy if delivered at 2 Gy per fraction. All the patients were evaluated by computed tomography (CT) scan in two months and fluorodeoxyglucose positron emission tomography (FDG-PET) every six months.

**Table 1:** Result of 10 cases Case 1 to 5 had radiation therapy for primary tumor in thymus. Case 6 to 10 had radiation therapy for multiple lesions of the metastatic tumor or relapse. The prescription dose varies according to the size of the tumor. The treatment was effective for both primary and metastatic tumor. Scc = Squamous cell carcinoma, CBDCA = Carboplatin, RTX = Paclitaxel, ADOC = Adriamycin + Cisplatin + Vincristine + Cyclophosphamide, PTV = planned tumor volume, Mo = months.

CASE	1	2	3	4	5	6	7	8	9	10
AGE	56	62	60	47	72	45	56	65	57	38
SEX	M	F	F	F	F	F	F	M	M	F
HISTOLOGY	Scc	Scc	Combined thymic epithelial tumor	Scc	Scc	Scc	Scc	Scc	Scc	Scc
MASAOKA STAGE	Ⅱ	Ⅳa	Ⅳa	Ⅳb	Ⅳb	Ⅳb	Ⅲ	Ⅳb	Ⅳa	Ⅳa
SURGERY	N/A	N/A	N/A	N/A	N/A	N/A	done	done	done	done
CHEMOTHERAPY	N/A	N/A	CBDCA+PTX	N/A	ADOC, CBDCA + PTX	CBDCA+PTX	N/A	CBDCA+PTX	done	CBDCA+PTX
FRACTIONS	8	10	10	10	7	1 to 6	12	1 to 8	3 to 5	5
PRESCRIPTION DOSE FOR PRIMARY TUMOR (cGy)	5000	4000	3100	3500	4200	N/A	N/A	N/A	N/A	N/A
PRESCRIPTION DOSE FOR METASTASIS OR RELAPSE (cGy)	N/A	N/A	N/A	N/A	2400 to 3200	200 to 4200	3800	2100 to 4500	3000 to 4000	5000
LOCATION OF TARGET LESIONS	Thymus	Thymus	Thymus	Thymus	Thymus, lymph nodes, pleura, and ribs	ribs, plevis, spine	mediastinum	pleura, ribs	lymph nodes, ribs	pleura
PTV (cm^3^)	83.8	354.5	460.8	747.3	198.1	6.1 to 41.2	646.3	0.2 to 112.8	5.8 to 81.0	22.4
OUTCOME	29 mo CR	9 mo PR	5 mo PR	13 mo PR	24 mo died	9 Mo Died	19 Mo CR	27 Mo PR	8 Mo PR	4 Mo PR

## Results

**Figure 2:**
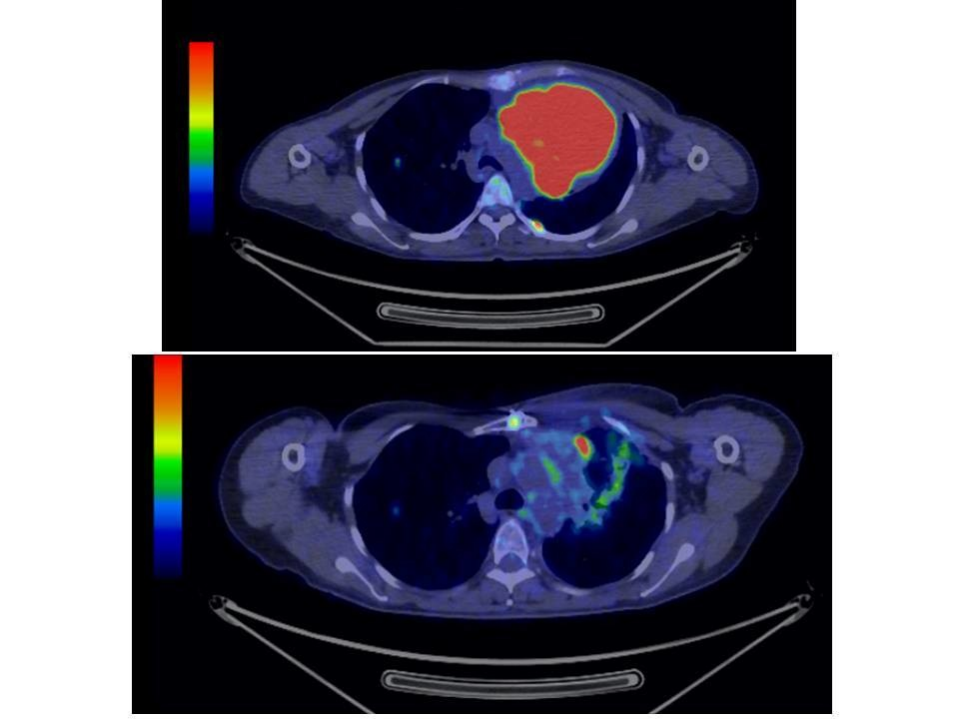
FDG-PET of Case 4 Before SBRT (top) and after SBRT (bottom).

The follow-up period was four to 29 months. All of the patients achieved partial remission (PR) in two months. For those who had FDG-PET in six months, the FDG intake was greatly decreased in the tumor as seen in Figure 2. Local recurrence was not seen at all. The patients of Stage II or III disease achieved complete remission (CR), and those in Stage IV showed progressive metastasis in the bones or the pleura after six months so that they continued SBRT for metastatic disease.

Case 1 was surgical indication, but he refused surgery because he had other complications, such as hepatocellular carcinoma. Fortunately, he achieved CR after SBRT, and no recurrence has been observed for 29 months. Case 7 had post-surgery relapse in the retroperitoneum and a large desmoid tumor in the right chest wall. Both tumors achieved CR after SBRT, and no recurrence has been observed for 19 months.

The other eight cases were Stage IV. Cases 3, 5, 6, 9, and 10 had failed in chemotherapy, and Cases 2 and 4 refused chemotherapy. Prior to SBRT, they had metastasis in the ribs, spine, or pleura that progressed in six months. However, all the tumors treated by SBRT achieved PR and FDG intake was greatly decreased in FDG-PET. Overall, tumor control was excellent with CyberKnife.

Case 8 was unique. He had surgery in 2005 but had multiple lung metastasis, pleural dissemination, and liver and bone metastasis. He had 10 operations of SBRT for metastatic disease from August 2013 to May 2015, followed by radiofrequency ablation for the metastatic liver cancer in June 2015. Adjuvant chemotherapy (Carboplatin and Paclitaxel, four courses) was done from August to November in 2015. FDG-PET in November 2015 showed PR. Whole body contrast CT scan in July 2016 did not show any recurrence at all. Now he is a long-time survivor of 11 years, even though he was in Stage IVb. This is a successful case of cancer management.

The side effects were minimal. Only four patients had mild radiation pneumonia that did not require treatment. Two patients, Cases 5 and 6, died of the progressive cancer. Adjuvant chemotherapy was recommended but was refused. Case 5 died of liver metastasis and Case 6 died of bone marrow metastasis; however, SBRT for bone metastasis was effective and reduced the pain.

## Discussion

Most of the patients were desperate when they first visited our office because they had been told that there is no optimal treatment for this cancer. Complete surgical resection is the gold standard to achieve cure; however, at an advanced stage, it is difficult and recurrence often occurs [9-10]. Radiation therapy has been considered effective as an adjuvant therapy for invasive lesions since thymic carcinoma is usually well radio-responsive [9,11].

All cases reached at least partial remission (PR) in two months with progression-free irradiated lesions and minimal radiation-related toxicity. CyberKnife SBRT was effective for the primary tumor, relapse, and metastasis of thymic cancer.

In the case of Stage IV disease, SBRT was effective only for the target tumor so that the pre-existing metastatic disease progressed after SBRT. Monden, et al. observed similarly that the recurrence rate after adjuvant radiotherapy for Stage III and IV was 20% while it was 50% in those not receiving irradiation, and most of the recurrences were outside the irradiated field [12]. In our cases, Stage IV disease had recurrences in the pleura, pericardium, and ribs, all of which were outside the irradiated field. The agenda is how to prevent metastasis or recurrence. Uematsu and colleagues proposed prophylactic entire hemithorax (or entire thorax) irradiation in addition to mediastinal irradiation [13]. The five-year relapse-free and overall survival rates were 100% and 96%; however, 13% of the patients had symptomatic radiation pneumonitis. Thus, the protocol of prophylactic irradiation needs improvement. Otherwise, SBRT should be combined with follow-up chemotherapy as it was in Case 8.

SBRT with CyberKnife is characteristic of less fraction and lower radiation effect to the surrounding organs. The radiation doses we used were smaller than the standard radiation therapy that Komaki, et al. recommended to use: radiation doses of 60-66 Gy for unresectable disease [14]. This could be the reason for the minimal toxicity by CyberKnife.

In our study, SBRT with CyberKnife was able to slow down the progress of cancer. What was even more beneficial to the patients was that each treatment course took less than 12 days and admission was not necessary. Some of the patients chose to stay in the hospital, but most of them commuted from home.

Fan, et al. reported the first instance of CyberKnife stereotactic ablative radiotherapy for Stage IVa thymic carcinoma [8]. Their case was successful with no recurrence and no radiation toxicity at 72 months’ follow-up. In our study, those who were able to receive SBRT for all the cancer lesions had long-time survival. If there are only a few metastatic lesions, complete remission is possible with CyberKnife SBRT.

## Conclusions

SBRT with CyberKnife was successful in patients with primary tumor, relapse, and metastatic lesions of thymic carcinoma. The duration of therapy was much shorter than standard radiation therapy, which made it easier for the patients of advanced cancer to receive the therapy.

SBRT with CyberKnife is an option for those who seek noninvasive alternative treatment. It preserves the patient’s quality of life and is beneficial even at the terminal stage. Combined with conventional surgery and chemotherapy, SBRT with CyberKnife has now been demonstrated to prolong patients’ lives.
